# Granule-stored MUC5B mucins are packed by the non-covalent formation of N-terminal head-to-head tetramers

**DOI:** 10.1074/jbc.RA117.001014

**Published:** 2018-02-13

**Authors:** Sergio Trillo-Muyo, Harriet E. Nilsson, Christian V. Recktenwald, Anna Ermund, Caroline Ridley, Lauren N. Meiss, Andrea Bähr, Nikolai Klymiuk, Jeffrey J. Wine, Philip J. B. Koeck, David J. Thornton, Hans Hebert, Gunnar C. Hansson

**Affiliations:** From the ‡ Department of Medical Biochemistry, University of Gothenburg, 40530 Gothenburg, Sweden,; the § Department of Biosciences and Nutrition, Karolinska Institutet, 14157 Huddinge, Sweden,; the ¶ School of Technology and Health, KTH Royal Institute of Technology, 14157 Huddinge, Sweden,; the ‖ Wellcome Trust Centre for Cell-Matrix Research, Faculty of Biology, Medicine, and Health, Manchester Academic Health Science Centre, University of Manchester, Manchester M139PT, United Kingdom,; the ** Institute of Molecular Animal Breeding and Biotechnology, Gene Center, Ludwig Maximilians University Munich, Hackerstrasse 27, 85764 Oberschleissheim, Germany, and; the ‡‡ Cystic Fibrosis Research Laboratory, Stanford University, Stanford, California 94305

**Keywords:** mucin, mucus, lung, secretion, EM, mucin bundle, regulated secretory pathway, submucosal gland

## Abstract

Most MUC5B mucin polymers in the upper airways of humans and pigs are produced by submucosal glands. MUC5B forms N-terminal covalent dimers that are further packed into larger assemblies because of low pH and high Ca^2+^ in the secretory granule of the mucin-producing cell. We purified the recombinant MUC5B N-terminal covalent dimer and used single-particle electron microscopy to study its structure under intracellular conditions. We found that, at intragranular pH, the dimeric MUC5B organized into head-to-head noncovalent tetramers where the von Willebrand D1–D2 domains hooked into each other. These N-terminal tetramers further formed long linear complexes from which, we suggest, the mucin domains and their C termini project radially outwards. Using conventional and video microscopy, we observed that, upon secretion into the submucosal gland ducts, a flow of bicarbonate-rich fluid passes the mucin-secreting cells. We suggest that this unfolds and pulls out the MUC5B assemblies into long linear threads. These further assemble into thicker mucin bundles in the glandular ducts before emerging at the gland duct opening. We conclude that the combination of intracellular packing of the MUC5B mucin and the submucosal gland morphology creates an efficient machine for producing linear mucin bundles.

## Introduction

The two mucins MUC5B and MUC5AC are large highly glycosylated polymers that are among the major macromolecular constituents in mammalian respiratory tract secretions. The MUC5AC mucin is normally made by surface goblet cells and the MUC5B mucin by submucosal glands (1). However, this differs among mammals because, for example, mice only have a few glands close to the larynx, whereas humans and pigs have on average one submucosal gland per square millimeter all the way down to about the 10th bronchial generation (2). The respiratory system is kept relatively free from inhaled bacteria and debris by the mucociliary clearance system. The majority of the cells in the conducting airways carry numerous cilia that are continuously beating at 10–20 Hz in the periciliary liquid (3). On top of the periciliary liquid layer is the airway surface liquid, which is transported cephalically by the cilia. We showed recently that pigs and most likely also humans, both with numerous submucosal glands, produce long linear mucus bundles that are transported cephalically (4). These mucus bundles are up to 30 μm thick when they emerge from the gland ducts and float in the airway surface liquid. The bundles have a central core made up by the MUC5B mucin. This core is further patchily covered by MUC5AC mucin, which is secreted from the goblet cells located in the distal ducts of the glands and the epithelial surface (4).

All four human gel-forming mucins (MUC2, MUC5B, MUC5AC, and MUC6) and the von Willebrand factor (VWF)^3^ have similar domain organizations. All form disulfide-bonded dimers in their C termini because of their cysteine knot domains (5). The N-terminal part of these molecules are made up of von Willebrand D assemblies built by VWDs in the order D1–D2–D'–D3 (5–7). VWD3 mediates the formation of covalent polymers of different types in the late secretory pathway. VWF and MUC5B form linear molecules as these form disulfide-bonded dimers in the N-terminal VWD3, whereas MUC2 forms trimers and thus net-like covalent structures (8–10). All mucins are stored in compact form in the regulated secretory granule of mucin-secreting cells because of the lower pH and high Ca^2+^ concentration in these granules. There is one single high-affinity Ca^2+^ ion–binding site in the MUC5B N terminus situated in the D3 domain and involved in calcium-dependent intermolecular association (9). In the case of MUC2, the N termini form concatenated ring structures on which the remaining part of the molecule and its C terminus stands (10). How the MUC5B mucin is stored in the cell is still not fully understood (9). Irrespective of the storage form, it is important that the storage is well organized, allowing smooth unfolding upon release from the mucin-producing cells. This release has to be made into a milieu that is sufficiently rich in bicarbonate to quickly raise the pH to remove the bound Ca^2+^ ions, as first suggested by Quinton (11) and as illustrated for the MUC2 mucin in the small intestine (12).

We have now studied how the MUC5B mucin is packed in the submucosal glands, allowing the formation of MUC5B mucin bundles. MUC5B N-terminal covalent dimers are shown to form non-covalent dimers, tetramers, in the low-pH, high-Ca^2+^ milieu found in the regulated secretory vesicles of mucus-secreting cells. The 3D structure of these tetramers was revealed by negative stain single-particle transmission electron microscopy (TEM). Each tetramer was made up of a covalent dimer, where one dimer is turned 180° and the von Willebrand D1–D2 domains of each hook into each other. When MUC5B is secreted, it meets the bicarbonate-rich fluid from the serous cells of the submucosal glands; there is dissociation of the coupled dimers and subsequent unfolding of the mucin and the formation of mucin bundles.

## Results

### The MUC5B N-terminal covalent dimer forms tetramers at low pH with Ca^2+^

**Figure 1.**
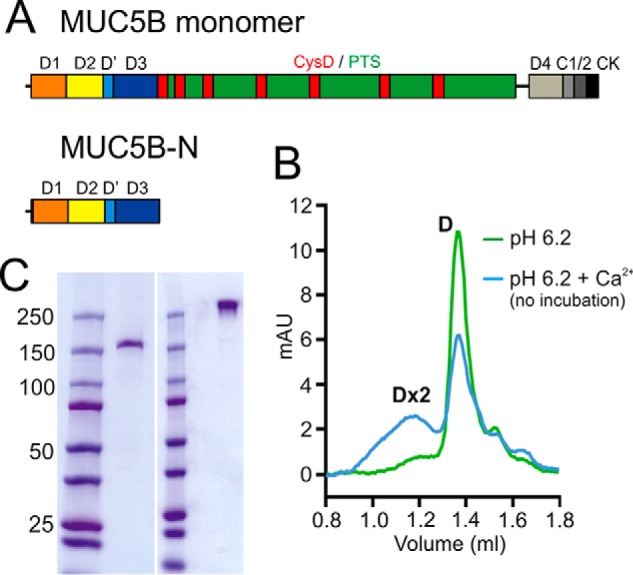
**Assembly of the MUC5B N termini at low pH and high Ca ^2+^.** *A*, the MUC5B monomer consists of the following domains: D1 (*orange*), D2 (*yellow*), D' (*light blue*), D3 (*blue*), D4 (*light gray*), CysD (*red*), the mucin domain (*PTS, green*), von Willebrand C (VWC), (C1 (*medium gray*) and C2 (*dark gray*)), and the cystine knot (*CK*) domain (*black*). The MUC5B-N recombinant protein included a His_6_ tag and the D1, D2, D', and D3 domains. *B*, MUC5B-N was analyzed by gel filtration chromatography on a Superose 6 3.2/300 column previously equilibrated in 50 mm MES (pH 6.2), 150 mm NaCl (*green*) or 50 mm MES (pH 6.2) 150 mm NaCl, 4 mm CaCl_2_ (*blue*). *D* means covalent MUC5B-N and *Dx2* its non-covalent dimer (tetramer). *mAU*, milliabsorbance units. *C*, SDS-PAGE stained by Coomassie Blue of the purified MUC5B-N under reducing (*left*) and non-reducing conditions (*right*). Molecular mass standards marked in kilodalton are shown to the *left* of the gel.

The MUC5B mucin produced by submucosal glands has an N-terminal part (MUC5B-N) containing the von Willebrand D1, D2, D', and D3 assemblies (Fig. 1*A*). These domains are known to be responsible for mucin oligomerization. When MUC5B-N was produced as a recombinant protein and studied by gel filtration, small angle X-ray scattering, and TEM at pH 7.4, a covalent dimer was observed (9). Because all polymeric mucins are known to be stored within secretory granules of mucin-producing cells at low pH and high Ca^2+^, the purified MUC5B-N was incubated at pH 6.2 with and without 4 mm Ca^2+^. Gel filtration showed a dimer (D) in the absence of Ca^2+^ ions. When Ca^2+^ was added, a mixture of dimeric and tetrameric (Dx2) molecules was observed (Fig. 1*B*). The MUC5B-N covalent dimer was migrating as a single band with a mass of over 250 kDa in its non-reduced form and also as a single band after reduction at its expected monomeric size of about 175 kDa (Fig. 1*C*). Together, this suggests that MUC5B-N is a covalent dimer that can further assemble into a non-covalent tetramer.

### 3D structure of the MUC5B-N tetramer (Dx2)

**Figure 2.**
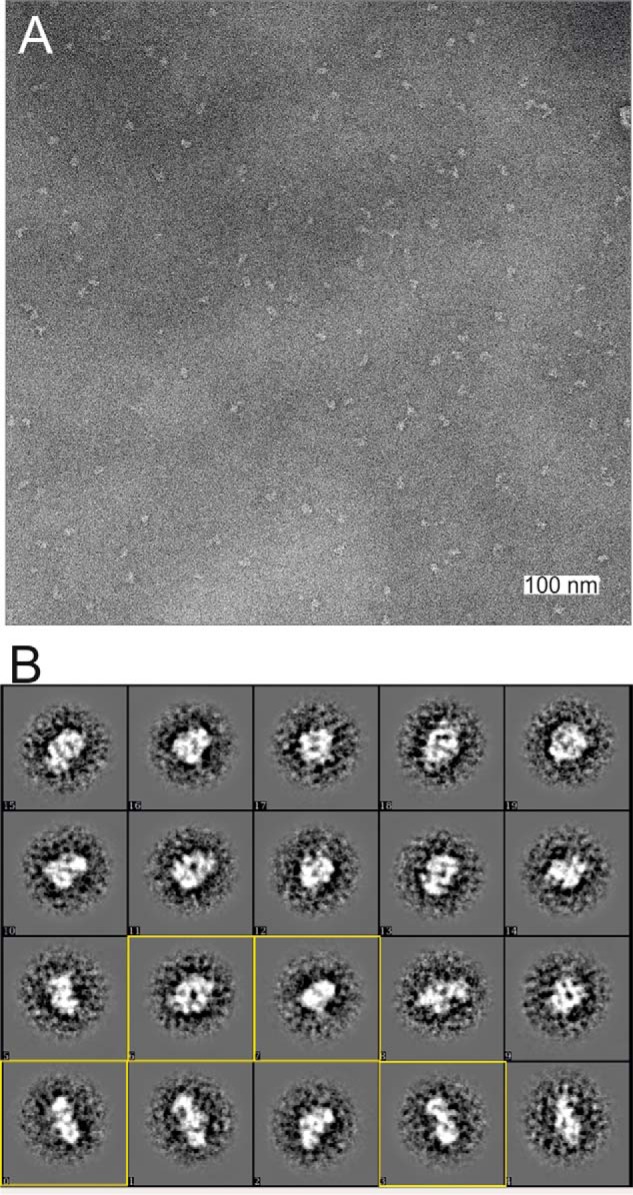
**Single-particle analyses of MUC5B-N Dx2.** *A*, an electron microscopy image of MUC5B-N oligomers. The sample was negatively stained in 2% uranyl acetate and imaged at room temperature in a Jeol JEM2100F electron microscope. *B*, MUC5B-N oligomers as 2D class averages of *boxed out regions* in negative stain. Side, tilted, and top views demonstrate an approximate 2-folded symmetry, as illustrated by *yellow boxes*. The size of each box is ∼40 nm.

**Figure 3.**
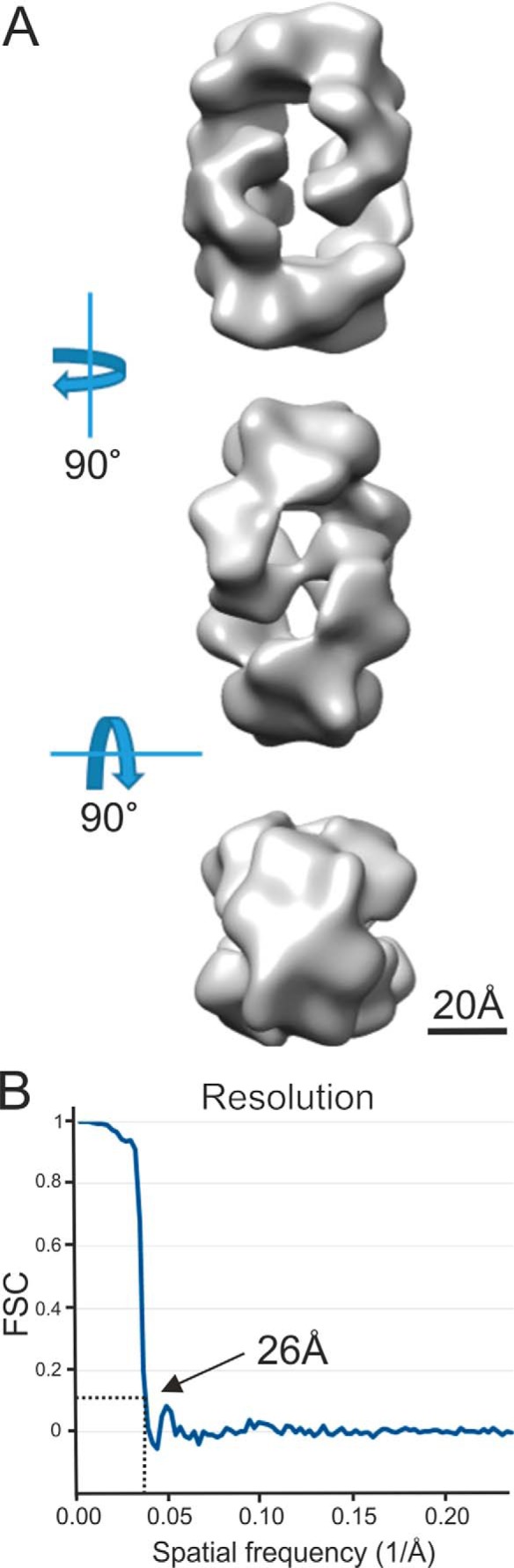
**Single-particle analysis of MUC5B-N Dx2.** *A*, 3D density map of the MUC5B-N oligomer with dihedral (D2) symmetry. The viewing directions are from the side parallel with the 2-fold axes and from the top. *B*, the Fourier shell correlation (*FSC*) curve between reconstructions produced by splitting the dataset in two halves. Both halves were reconstructed separately. The resolution (26 Å) for the reconstructed 3D density map was calculated from the curve at FSC = 0.143 (*dotted line*).

To further confirm the nature of the MUC5B-N Dx2 molecule as formed at pH 6.2 and 4 or 20 mm Ca^2+^, it was studied by TEM using negative staining. The Dx2 molecules were relatively unstable, and a short glutaraldehyde incubation was used to stabilize them before adding the sample to the TEM grid. The single particles observed were relatively homogenous, allowing their collection (Fig. 2*A*). Reference-free 2D classification using 4056 particles representing different orientations revealed 2-fold symmetry in some of the classes (Fig. 2*B*). This is in agreement with biochemical data as well as earlier obtained results from single-particle reconstructions (9). 3D map reconstruction and refinement were performed using D2 symmetry, as a reconstruction without imposed symmetry (C1) suggested a D2 symmetry (Fig. S1). The D2 symmetry produced a map at a final resolution of 26 Å based on 3D Fourier shell correlation following the gold standard FSC procedure (Fig. 3). The stain covered a complex that could be fitted into a box of ∼135 × 135 × 95 Å^3^ at a contour level, which clearly depicted the substructure of the protein.

**Figure 4.**
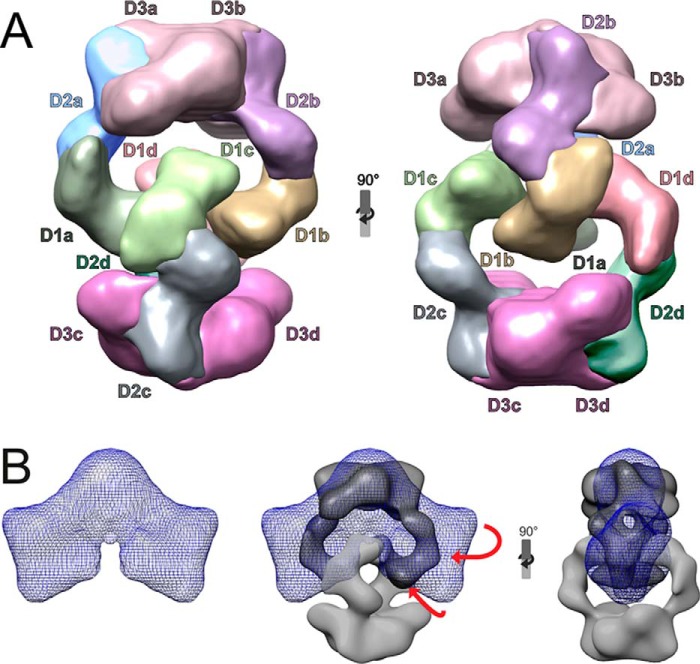
**3D structure of MUC5B-N Dx2.** *A*, density map of MUC5B-N incubated at pH 6.2 with 20 mm Ca^2+^. Domains are color-marked *D1*, *D2*, and *D3*, with subheadings *a–d* for each of the four MUC5B-N molecules. The colored model is also shown rotating in two planes in Movie S1. In the structure recorded at pH 6.2 with Ca^2+^, the D1–D2 arms are turned inward and, by this, hook the two dimers together. *B*, comparison of the 3D structure of MUC5B-N Dx2 at pH 6.2 with 20 mm Ca^2+^ with the MUC5B-N at pH 7.4 without Ca^2+^ (the “fan”-like model to the *left*, from Ref. 9). The two models (*left* and *A*) are superimposed in the *center* and *right* (turned 90°).

The domains of the obtained structure were analyzed based on previous observations of the MUC2 N-terminal VWD D3 trimer (10, 13) and the MUC5B-N at pH 7.4 (9). The observed images revealed molecules made up by two of these dimers held together covalently at VWD D3a–D3b and D3c–D3d (Fig. 4*A*). One of the dimers was turned 180°, and the two arms of each dimer held the Dx2 tetramer together. The obtained resolution allowed assignment of the different VWD domains and showed that the four arms hooked into each other. Each of the MUC5B-N molecules most likely had an arm made up by the VWD1 and VWD2 domains interacting non-covalently with the two arms of the other covalent dimer (see Fig. 4*A*, where individual domains are color-coded). Comparing the positions of the VWD1 and VWD2 arms with that of the MUC5B-N determined at pH 7.4 (9) suggested that the arms had folded inwards and, by this, hooked the two dimers to each other (Fig. 4*B*). Looking from the side, the two dimers were turned about 50° toward each other. The tetrameric model is further illustrated by rotation in Movie S1.

Further proof of the model was obtained by separating the glutaraldehyde-stabilized Dx2 tetramer by electrophoresis (Fig. S2) and digesting the tetramer with Glu-C protease, followed by analyzing the obtained peptides by nanoLC-MS. In addition to cross-linked peptides in the dimer only, one peptide from the Dx2 sample showed a cross-linked peptide between amino acids 86 to 592 found in the D1 and D2 domains, respectively (Fig. S3*A*). This location is in accordance with the predicted model shown in Fig. 4*A*.

### The MUC5B N-terminal tetramers form linear assemblies

**Figure 5.**
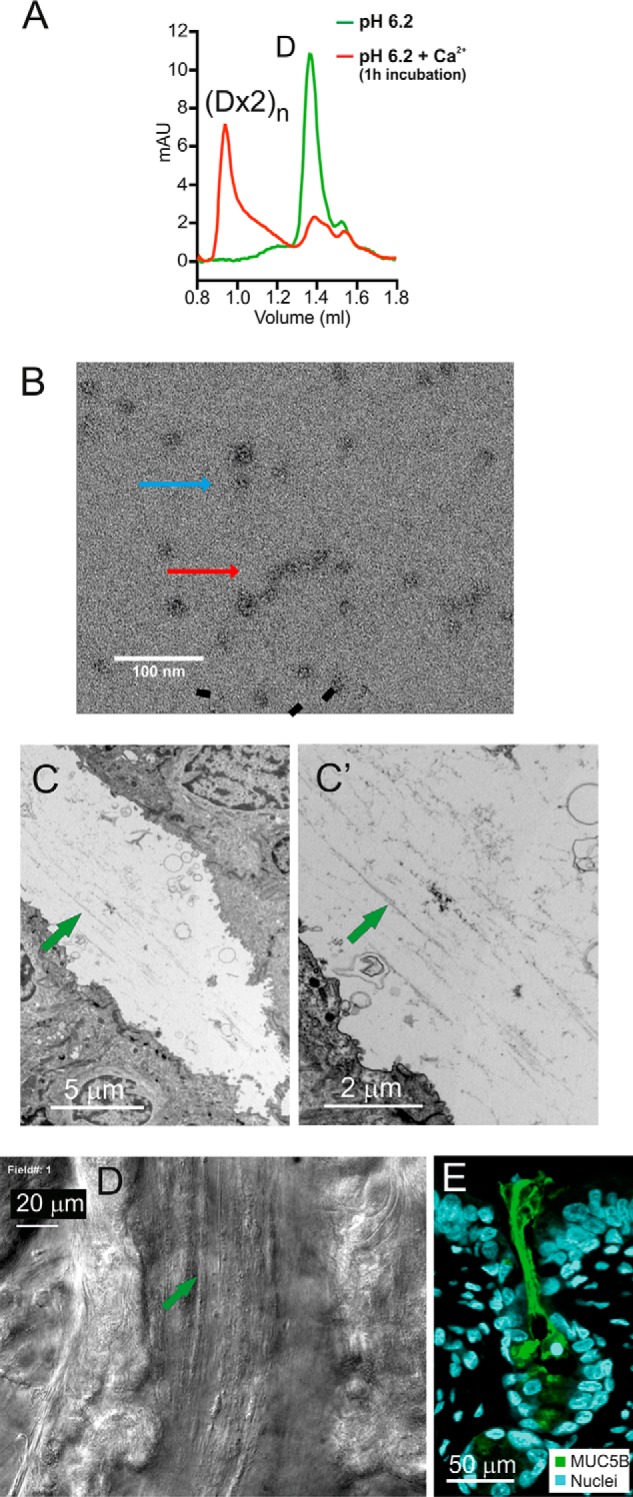
**Packing of the MUC5B mucin in the intracellular storage granule and release into the gland lumen.** *A*, MUC5B-N was analyzed by gel filtration chromatography on a Superose 6 3.2/300 column previously equilibrated in 50 mm MES (pH 6.2) and 150 mm NaCl (*green*) or incubated for 1 h in 50 mm MES (pH 6.2), 150 mm NaCl, and 4 mm CaCl_2_ (*red*). MUC5B-N without calcium showed one peak corresponding to the dimer; with calcium incubation, most of the protein eluted in the void volume corresponding to high-order oligomers, suggesting heterogeneous oligomerization (*(Dx2)_n_*). *mAU*, milliabsorbance units. *B*, MUC5B-N incubated at pH 6.2 with 20 mm Ca^2+^ and viewed by negatively stained TEM. *Blue arrow*, single Dx2 particle; *red arrow*, linear arrangement of particles (*(Dx2)_n_*). *C*, proximal gland duct filled with linear material (*green arrow*), likely MUC5B mucin bundles (TEM). *C'*, *C* at higher magnification. *D*, mucus strands moving through a human submucosal gland tract, frame from Movie S2. *E*, immunofluorescence in a paraffin section from pig trachea. Shown is MUC5B in bundles secreted from the submucosal gland (*green*). The staining was repeated in sections from five different pigs.

When MUC5B-N was incubated at pH 6.2 with 4 mm Ca^2+^ for 1 h, larger complexes were formed, as shown by the broad early peak and void volume–eluting peak upon gel filtration (Fig. 5*A*). In addition to the tetrameric particles, TEM in negative stain after incubation with 20 mm Ca^2+^ (pH 6.2) showed structures that lined up and looked like linear assemblies (Fig. 5*B*). Although these structures were very heterogeneous in length, one can suggest that they are linearly arranged.

### The submucosal glands form linear bundles containing the MUC5B mucin

When the packed mucin is secreted from mucus-producing cells, it has to unfold and expand. This unwinding of mucin has not been possible to observe in tissue, but further up in the duct of the piglet submucosal gland duct, linear threads were observed using TEM (Fig. 5, *C* and *C'*). These threads probably reflect full-length MUC5B polymers. When the ducts of single human submucosal glands were observed by time-lapse video microscopy, threads moving with the flow were visualized (Fig. 5*D*, *arrow*, and Movie S2). The liquid flow as shown by particle flow seemed faster than the strands, suggesting that the flow could generate a pulling force that pulled out and helped to unfold the mucin. The threads gathered and became thicker, and in the gland opening, thick mucin bundles could be observed after staining with an anti-MUC5B antiserum (Fig. 5*E*). Linear bundles of mucus coming out of the submucosal gland ducts has been observed previously by scanning EM (4) and correlates well with the unfolding and assembly of the mucin bundles predicted here.

## Discussion

### The MUC5B N-terminal tetramers can form linear side-to-side polymers in the intracellular storage granule

**Figure 6.**
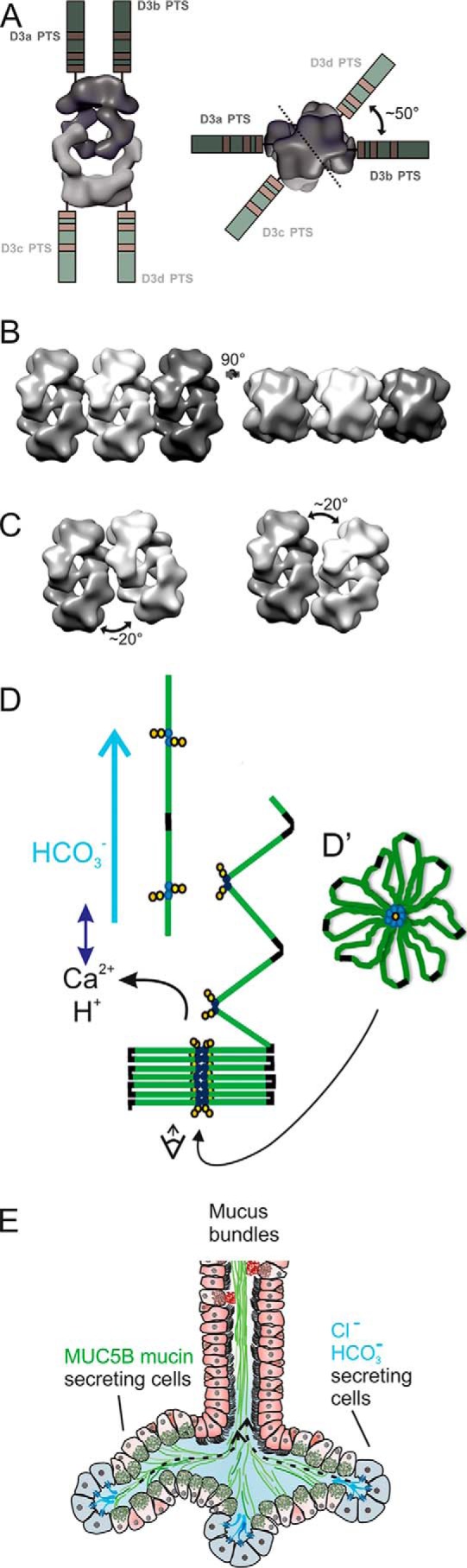
**The submucosal gland is the ultimate machine for shaping mucus bundles made of MUC5B.** *A*, the remaining C-terminal parts of the MUC5B mucin, including the extended mucin domains and the C terminus, extends out from the D3 domain in two possible variants, parallel to the *y* axis (*left*) or parallel to the *x* axis (*right*). *B*, a possible packing of the Dx2 tetramer as 3D models inside the cell at the pH of the granule. *C*, the packing illustrated in *B* is suggested to have a better fit if each tetramer is tilted 20°, as can be suggested from the picture in Fig. 5*B. D*, drawing illustrating MUC5B unfolding with domains color-coded as in Fig. 1*A* and the effect of HCO_3_^−^ flow (pH increase, Ca^2+^ detachment, pulling). *D'*, model of MUC5B intracellular packing, modified from Ref. 9, with domain colors as in Fig. 1*A. E*, schematic of a submucosal gland. The serosal cells at the bottom of the gland are *light blue* and secrete chloride and bicarbonate ions to generate a flow as marked by *dashed arrows*. Mucus-secreting cells are more proximal to the gland opening and secrete MUC5B (*green*).

The MUC5B mucin is assembled into linear polymers by C-terminal disulfide-bonded dimers in the endoplasmic reticulum and further to N-terminal disulfide-bonded dimers in the trans-Golgi network secretory vesicles (9, 14). The mucin stored in the mucus granule has to be packed in a manner that allows it to unfold and expand in a controlled way upon secretion. This is accomplished by the decreased pH and increased Ca^2+^ concentration in the late secretory pathway, where binding of Ca^2+^ triggers a conformational change in the N terminus of the mucin. For the MUC2 mucin, this has been explained by the ability of its N terminus to form ring-like structures, allowing it to expand into large nets upon secretion (10). Using the MUC5B N terminus, we have now been able to show how the structural arrangement works for this mucin. At lower pH and in the presence of Ca^2+^ ions, two covalent dimers form head-to-head non-covalent tetramers (Dx2). This is triggered by a conformational change where the VWD1–VWD2 parts are suggested to bend inward and, by this, interact. MUC5B-N is only a minor part of the full-length MUC5B mucin (Fig. 1*A*). The mucin domains and the MUC5B C terminus has to extend out from the VWD3 of the N terminus. Two possible directions are suggested in Fig. 6*A*. Both predict a central N-terminal core with the mucin domains pointing outward and with the C terminus farthest away from this core.

The tetramers are further interacting as they form linear assemblies, as observed by TEM in Fig. 5*B*. Of the two variant directions shown in Fig. 6*A*, the left one is more likely, as it opens the side of the tetramer for further interactions and packing. The model to the right suggests interactions of VWD3 domains, something that is not likely, as this part of the N terminus is less affected by the low pH–Ca^2+^ conformational change. When the surfaces exposed in Fig. 6*A*, *left model*, were analyzed in 3D, a relatively tight sideways packing could be obtained (Fig. 6*B*). This interaction was even more complementary when the tetramers were tilted 20° relative each other (Fig. 6*C*). As the MUC5B-N model is symmetric, this will allow bending back and forth without forming a circular assembly. We do not have any more detailed proof of this model, but the bending is in relative accordance with the TEM picture in Fig. 5*B*, where the particles are linearly arranged, but not in a perfect linear way, as each particle seems to be slightly tilted relative each other. This means that the “linear arrangement” is not identical to the straight spirals observed for the VWF (15). However, it is principally similar, as the C-terminal parts of both VWF and MUC5B point outward from a central core.

Combining all of our results, this could suggest that MUC5B is intracellularly packed, as suggested in Fig. 6*D*, *bottom*: a central N-terminal core with the mucin and C-terminal domains pointing outward. Interestingly, such structures have already been observed. In a TEM micrograph from saliva, where the major mucin is MUC5B, a view from the end of such an assembly has been observed by Kesimer *et al.* (Fig. 6 in Ref. 16). An adapted model with the used colors is shown in Fig. 6*D'*. More recently, the same group has also shown the predicted side view in an atomic force microscopy (AFM) image (Fig. 3*a* in Ref. 17). In both cases, the packed mucin can be suggested to have escaped unpacking and to show the intracellularly stored form. Together, the observations by us and others suggest how the MUC5B mucin may be packed in the granule of mucin-producing cells.

### The submucosal gland is a machine for forming linear MUC5B bundles

The submucosal glands have an anatomical arrangement ideal for the generation of linear mucin strands and bundles (Fig. 6*E*). Bicarbonate ions have been shown to be important for unpacking the granule-stored mucins, as bicarbonate raises the pH and removes Ca^2+^ ions from the high-affinity binding site on the N-terminus of the mucin (11, 12). For MUC5B, this means opening the intertwining hooks formed by the VWD1–VWD2 clamp (Fig. 4). In the submucosal glands, a fluid flow is generated by the most distal cells, the serous cells (Fig. 6*E*) (2, 18). These cells have abundant CFTR channels that generate a liquid flow by secreting chloride and bicarbonate ions. This flow passes by the more proximally placed MUC5B mucin–producing cells. In these cells, the MUC5B mucin is packed in the granule because of low pH and Ca^2+^. When the mucins are secreted, they immediately hit the bicarbonate-rich flow. This will remove the Ca^2+^ ions from binding the MUC5B and release the clamp that assembles the stored mucin. This process and the directed flow will allow the MUC5B mucin to unfold and extend, as suggested in the model in Fig. 6*D*. The bicarbonate-rich fluid flow can mediate unfolding, similar to that generated by the blood flow that unwinds the VWF (19–21).

Important for the expansion and linearization of mucins upon secretion is the central, highly glycosylated, rod-like, densely glycosylated mucin domain that will bind water and expand the mucin when allowed. The same mucin can have different *O*-glycans, as observed in different species and locations, suggesting that it is the general water-binding capacity of the glycans that is important rather than the specific structure of the glycans.

Linear threads can be observed by TEM in the gland ducts, and using video recordings, we could observe these threads moving. In the most proximal part of the glands and in their openings, bundles stained for MUC5B were observed. The thickness of the bundles suggests that a number of MUC5B polymers interact laterally. Appearing at the submucosal gland opening into the trachea, thick bundles with a diameter of 25–30 μm were observed, suggesting that each bundle comprises more than 1000 polymers (4). The secreted bundles will float on the normal thin air surface liquid of less than 10 μm. The formation of bundles containing multiple polymers held together by lateral interaction has also been observed for the von Willebrand factor (22).

Pigs and humans have numerous submucosal glands, from the nasal cavities through the trachea and bronchi down to airways of ∼2-mm diameter (2). As shown here, these glands are, by their organization, efficient machines making linear MUC5B mucin bundles. These thick bundles are transported cephalically and ventrally by the beating cilia, moving bacteria and debris to the larynx (4, 23). This is different from rodents, which largely lack glands and in which the surface cells do not secrete MUC5B in bundles. Instead, mice can be observed to have mucus shaped like “clouds.”

We now have structural information of how three of the five human proteins with N-terminal D1–D2–D'–D3 domain organization, the VWF, MUC2, and MUC5B, are packed in the intracellular storage granule. Interestingly, despite their high similarities in amino acid sequence, they form different supramolecular assemblies in the low-pH, high-Ca^2+^ milieu. The MUC2 mucin forms concatenated ring structures largely built by six-membered rings (10). Upon release, MUC2 forms large net-like sheets. VWF is stored as long stiff spirals that form the granule into Weibel–Palade bodies (15). Upon secretion, these spirals slowly unwind by the pulling forces generated by the blood flow (19, 20). Finally, as shown here, the MUC5B mucin also forms linear assemblies, but in this case by every second covalent dimer turned 180° and hooked into each other. Upon release, these assemblies are pulled out into linear polymers that interact laterally to form thick mucin bundles. Further understanding of the molecular details of these differences must await more detailed structural information, something that cryo-EM might provide.

## Experimental procedures

### Production and analysis of MUC5B-N

The recombinant MUC5B N-terminal (GenBank accession number NM_002458, residues 26–1304) construct was expressed with an N-terminal His_6_ tag using the mammalian episomal expression vector pCEP-His (9). CHO-K1 cells were grown in Iscove's modified Dulbecco's medium with 10% FBS (Lonza) at 37 °C with 5% CO_2_. Transfection of CHO-K1 cells with pCEP-His-MUC5B-N was performed using Lipofectamine 2000 (Invitrogen) in 6-well plates in accordance with the manufacturer's instructions. Puromycin (Life Technologies, 10 μg/ml) was added for selection 2 days after transfection. Generation of stably producing clones was performed by seeding transfected cells into 9-cm Petri dishes to obtain separate colonies and then screening for secreted recombinant protein among isolated clones grown in a 96-well plate. A high-producing clone was adapted to grow in suspension using ProCHO-4 with 1×ProHT and 4 mm l-glutamine (Lonza) with 10 μg/ml puromycin in 250-ml spinner flasks. The FBS concentration was initially 2% and could be reduced stepwise to 0% after the cells reached 1 × 10^6^/ml in the presence of 2% FBS. The adaptation took 2 months. Collection of 1 liter of cell supernatant with recombinant MUC5B-N protein was performed in spinner flasks. At harvest, the cell suspension was centrifuged at 200 × *g* for 5 min at room temperature, and the supernatant was stored at 4 °C with 0.05% NaN_3_.

The spent culture medium containing recombinant protein was filtered (0.65 + 0.45 μm Sartobran 300 capsule, PALL) and concentrated by tangential flow filtration (Pellicon^TM^-2 system, Millipore) with a 30-kDa PLCGC filter. A buffer change was performed when the volume of the concentrate was reduced to 300 ml by addition of 300 ml PBS with 0.05% NaN_3_, followed by another reduction of the volume to 300 ml. The procedure was repeated five times, and the concentrate volume was finally reduced to 200 ml, which was filtered (Durapore® membrane filter, 0.22 μm GVWP, Millipore).

MUC5B-N was purified by nickel affinity chromatography using a 5-ml HiTrap chelating HP column (GE Healthcare) eluted with a gradient of 10–300 mm imidazole in 20 mm Tris and 150 mm NaCl (pH 7.4), followed by size fractionation on a Superose 6 10/300 column (GE Healthcare) eluted in 20 mm Tris and 150 mm NaCl (pH 7.4) using an ÄKTA purifier (GE Healthcare). For size exclusion chromatography analysis, MUC5B-N was incubated for 0 or 1 h at room temperature in 50 mm MES, 150 mm NaCl, pH 6.2 or 50 mm MES, 150 mm NaCl, and 4 mm CaCl_2_ (pH 6.2) and then applied to a Superose 6 3.2/300 column (GE Healthcare) previously equilibrated in the same buffer using an Ettan LC (GE Healthcare).

### Single-particle analysis and reconstruction

Samples (10–15 μg/ml) in 20 mm MES (pH 6.2), 20 mm CaCl_2_, and 150 mm NaCl were incubated at room temperature overnight and then fixed in 0.6% glutaraldehyde for 4 min. Aliquots (4 μl) were adsorbed onto glow-discharged continuous carbon-coated copper grids (400 mesh, Analytical Standards) for 5 min. The grids were subsequently blotted with filter paper, washed with two drops of milli-Q water, and negatively stained with one drop of 2% (w/v) uranyl acetate for 45 s before final blotting and air-drying.

Data were collected using a Jeol JEM2100F field emission gun transmission electron microscope operating at 200 kV. Micrographs were recorded at a magnification of ×50,000 and 1.3- to 2.0-μm defocus. The selected magnification resulted in a pixel size of 2.08 Å at the specimen level. Images were recorded on a 4000 × 4000 CCD camera (Tietz video and imaging processing system). The total electron dose was in the range of 8–13 e^−^ per Å^2^. A total of 74 images were recorded.

Images were imported to EMAN2 (version 2.12) (24). Defocus, particle separation, and amplitude contrast were evaluated with e2evalimage.py. Single particles, 4456, in different orientations were selected from the images using e2boxer.py in swarm mode. False positives, such as aggregated particles and stain artifacts, were discarded. For each image, the contrast transfer function parameters were estimated on boxed-out regions, 192 × 192 pixels, (containing particles) using the e2ctf.py program. Reference-free 2D classification was performed using 4056 phase-flipped particles with e2refine2d.py. 2D classes representing different orientations were selected for the initial model generation with e2initialmodel.py. A 2-fold symmetry was revealed. This was in agreement with biochemical data as well as results obtained earlier from single-particle reconstructions (9). 3D refinement was performed using e2refine_easy.py applying D2 symmetry aiming at a resolution of 26 Å. The first 3D refinement was performed with an angular step of 9.0 degrees. A second 3D refinement was performed with an angular step of 6.4 degrees, using the final map from the first refinement above as input. The determination of the resolution, 26 Å, was based on 3D FSC = 0.143 (25), following the gold standard FSC procedure implemented in EMAN2 (26). 70% of the total amount of collected particles contributed to the final map. The 3D map has been deposited with EMDB accession code EMD-4296.

A 3D refinement without imposed symmetry (C1) was also calculated to prove that the molecule has a 2-fold symmetry (Fig. S1). This map was generated with wider angular steps than the corresponding map with imposed D2 symmetry. The first 3D refinement was performed with an angular step of 13°. The second refinement was performed with an angular step of 10°, using the final map from the first refinement as input. The obtained resolution was 30 Å. The resolution of the map was estimated using 3D FSC with a threshold of 0.143. The final map was low pass–filtered to 50-Å resolution.

### Piglet airway preparation

WT piglets were euthanized under ketamine (Ursotamin®, Serumwerk Bernburg, Bernburg, Germany) and azaperone (Stresnil®, Elanco Animal Health, Bad Homburg, Germany) anesthesia by intracardial injection of T61® (Intervet GmbH, Unterschleissheim, Germany) according to the manufacturer's instructions. Animals were then dissected, and the airways including the larynx, trachea, and lungs, were explanted in total and immersed in chilled Perfadex® solution (XVIVO Perfusion, Gothenburg, Sweden) that had been adjusted to pH 7.2 with 1 m Tris. All connective and pulmonary tissues surrounding the larynx, trachea, and bronchial tree were then removed using anatomical forceps and surgical scissors. Airways were bathed in adjusted Perfadex® solution throughout the whole procedure. The prepared airways were then transferred to a 50-ml plastic tube (Falcon, BD Biosciences) filled with fresh pH-adjusted Perfadex® solution and shipped under chilled conditions overnight to the University of Gothenburg. Animal experiments were approved by the University of Munich, Germany, and Jordbruksverket, Jönköping, Sweden.

### Immunofluorescence staining of fixed sections

Pig airway tissue was fixed in 4% formalin, embedded in paraffin, and cut in 4-μm-thick sections that were dewaxed using xylene substitute (Sigma) and hydrated. For immunohistochemistry, antigen retrieval was performed by microwave heating in 0.01 m citric buffer (pH 6). Sections were blocked for 60 min with Tris-buffered saline (TBS) with 3% donkey serum and 0.1% Triton X-l00. The primary antibody MUC5B (a kind gift from M. Kesimer, University of South Carolina, Chapel Hill, SC) in blocking solution was incubated overnight at 4 °C and washed in TBS three times for 5 min before adding donkey anti-rabbit Alexa 555 (Thermo Fisher Scientific, Waltham, MA). Nuclei were counterstained with Hoechst 34580 (Thermo Fisher Scientific). Slides were washed in TBS three times for 5 min and mounted with Prolong Gold mounting medium (Thermo Fisher Scientific). Images were acquired with an upright LSM 700 Axio Examiner 2.1 confocal imaging system (Carl Zeiss, Oberkochen, Germany).

### Electron microscopy

Pig airway tissue was fixed in Karnovsky's fixative (2% paraformaldehyde, 2.5% glutaraldehyde in 0.05 m sodium cacodylate buffer (pH 7.2)) for 24 h, followed by preparation for TEM by sequential staining using 1% OsO_4_ for 4 h, 1% tannic acid for 3 h, and 1% uranyl acetate overnight. Samples were dehydrated and embedded in epoxy resin (Agar 100, Agar Scientific, Stansted, UK). Electron microscopy was conducted on 50-nm sections cut using an Ultracut E (Reichert, New York, NY) microtome and collected on mesh copper support grids. The sections were contrasted using lead citrate and tannic acid, and images were acquired using a Leo 912 Omega lanthanum hexaboride gun TEM (Carl Zeiss) at 120 kV and a MegaView III CCD camera (SiS, Münster, Germany).

### Video recording of gland secretion

Tracheal trimmings from a lung transplant donor were obtained within 4 h of surgery and pinned out in cold buffer (115 mm NaCl, 2.4 mm K_2_HPO_4_, 0.4 mm KH_2_PO_4_, 25 mm NaHCO_3_, 1.2 mm MgCl_2_, 1.2 mm CaCl_2_, 10 mm glucose, and 1.0 μm indomethacin (pH 7.4)). The mucosae and attached glands were dissected from cartilage and pinned out mucosal side down, and a suitable gland was dissected from the tissue and pinned onto a chamber made from a thin coating of Sylgard on a microscope slide with a surrounding wall of Sylgard ∼2 mm thick. Glands were secured with cactus spines and microdissected with fine forceps, needles, and iridectomy scissors until relatively clean and flat portions of gland were available for imaging. The chamber was placed on the stage of a Nikon Eclipse E600FN series microscope equipped with differential interference contrast (DIC) optics and a ×40 water immersion objective. The chamber was continuously perfused with buffer (115 mm NaCl, 2.4 mm K_2_HPO_4_, 0.4 mm KH_2_PO_4_, 25 mm NaHCO_3_, 1.2 mm MgCl_2_, 1.2 mm CaCl_2_, 10 mm glucose, and 1.0 μm indomethacin (pH 7.4)). The chamber temperature (37 °C) was maintained with a TS-4 Peltier effect temperature controller. Digital images were taken with a Retiga-1300, cooled, 12-bit, color Bayer Mosaic CCD camera with an RGB liquid crystal color filter module run by a computer running Compix image capture and analysis software.

## Author contributions

S. T.-M., H. E. N., A. E., J. J. W., D. J. T., and G. C. H. conceptualization; S. T.-M., H. E. N., C. V. R., A. E., L. N. M., J. J. W., H. H., and G. C. H. data curation; S. T.-M., H. E. N., C. V. R., A. E., L. N. M., J. J. W., P. J. K., H. H., and G. C. H. formal analysis; S. T.-M., H. E. N., A. E., D. J. T., and G. C. H. validation; S. T.-M., H. E. N., C. V. R., A. E., L. N. M., J. J. W., and G. C. H. investigation; S. T.-M., H. E. N., A. E., L. N. M., J. J. W., and G. C. H. methodology; S. T.-M., H. E. N., A. E., C. R., L. N. M., A. B., N. K., J. J. W., P. J. K., D. J. T., H. H., and G. C. H. writing-review and editing; A. E., J. J. W., and G. C. H. writing original draft; G. C. H. resources; N. K., J. J. W., D. J. T., and G. C. H. funding acquisition; G. C. H. supervision; G. C. H. project administration.

## Supplementary Material

Supporting Information

## Footnotes

3 The abbreviations used are:

VWF: von Willebrand factor
TEM: transmission electron microscopy
VWD: von Willebrand D domain
FSC: Fourier shell correlation
CHO: Chinese hamster ovary
FBS: fetal bovine serum
TBS: Tris-buffered saline
MUC5B-N: N-terminal part of MUC5B
CCD: charge-coupled device.
