# Supplementary material for: Granule-stored MUC5B mucins are packed by the non-covalent formation of N-terminal head-to-head tetramers
Source: J Biol Chem. 2018 Feb 13;293(15):5746–54. doi: 10.1074/jbc.RA117.001014 (PMC5900763; doi:10.1074/jbc.RA117.001014)
Supplement: Supporting Information [file supp_293_15_5746__index.html]

Granule-stored MUC5B mucins are packed by the non-covalent formation of N-terminal head-to-head tetramers — Structure of stored MUC5B mucin — Granule-stored MUC5B mucins are packed by the non-covalent formation of N-terminal head-to-head tetramers — Structure of stored MUC5B mucin — Supporting Information 

# Granule-stored MUC5B mucins are packed by the non-covalent formation of N-terminal head-to-head tetramers

## Supporting Information

- Supplemental material - Supplemental methods, Figs S1-S3 and legends to Movie S1-S2
- Movie S1 - Figure 4A animated
- Movie S2 - ucin bundles in submucosal gland
