# Supplementary material for: Granule-stored MUC5B mucins are packed by the non-covalent formation of N-terminal head-to-head tetramers
Source: J Biol Chem. 2018 Feb 13;293(15):5746–54. doi: 10.1074/jbc.RA117.001014 (PMC5900763; doi:10.1074/jbc.RA117.001014)
Supplement: Supporting Information [file supp_RA117.001014_133986_2_supp_72094_p3twsn.pdf]

Supplementary material to

Granule-stored MUC5B mucins are packed by the non-covalent formation of N-terminal head-to-head tetramers

Supplementary Movies S1-S2 (separate video files)

Supplementary experimental procedures

Supplementary Figures S1-S3

Supplementary Movies

MOVIE S1. The model shown in Fig. 4A shown rotating followed by a 90° tilt.

MOVIE S2. Mucus strands moving through the submucosal gland tract. Flow of mucus and liquid in the collecting duct of a human tracheal submucosal gland, at steady state after ~1 h of stimulation with 10 µM forskolin at 36.6ºC. Images taken at 10 s intervals.

Supplementary experimental procedures

In-gel digestion and Mass spectrometric analyses of Cross-linked peptides.

Glutaraldehyde-treated MUC5B mono-, di- and tetramers were separated via SDS-PAGE on a 3 – 8% gradient gel followed by staining with Coomassie brilliant blue. Protein bands of interest were cut into ≈ 1 mm^3 pieces and destained with 50% acetonitrile. Gel pieces were dried in a vacuum centrifuge and digested with Glu-C (Promega) in 20 mM phosphate buffer pH 7.0 for 12 h at 37˚C. Peptides were extracted followed by removal of salts via in-house stage tips as recently described (1). MS analysis was performed as previously described (2) and cross-linked products were identified using the protein prospector software tool (http://prospector2.ucsf.edu/prospector/mshome.htm; version 5.20.0). Mass spectrometry data files were searched for cross-linked peptides using the following settings: mass tolerance of the precursor ion of 5 ppm; tolerance for fragment ions 200 ppm; Glu-C as cleaving enzyme with up to 3 missed cleavages allowed; carbamdiomethylation of Cys as fixed modification; oxidized methionine as variable modification; gain of 64.0313 for cross-linked products. Fragmentation spectra of putative candidates were further analyzed manually.

References

1. Recktenwald, C. V. and Hansson, G. C. (2016) The reduction-insensitive bonds of the MUC2 mucin are isopeptide bonds. *J. Biol. Chem.* **291,** 13580-13590

2. Johansson, M. E. V., Jacobsson, H. E., Holmén-Larsson, J., Schütte, A., Ermund, A., Rodriguez-Pineiro, A., Arike, L., Wising, C., Svensson, F., Bäckhed, F., and Hansson, G. C. (2015) Normalization of the host intestinal mucus systems requires long-term colonization. *Cell Host Microbe* **18,** 582-592

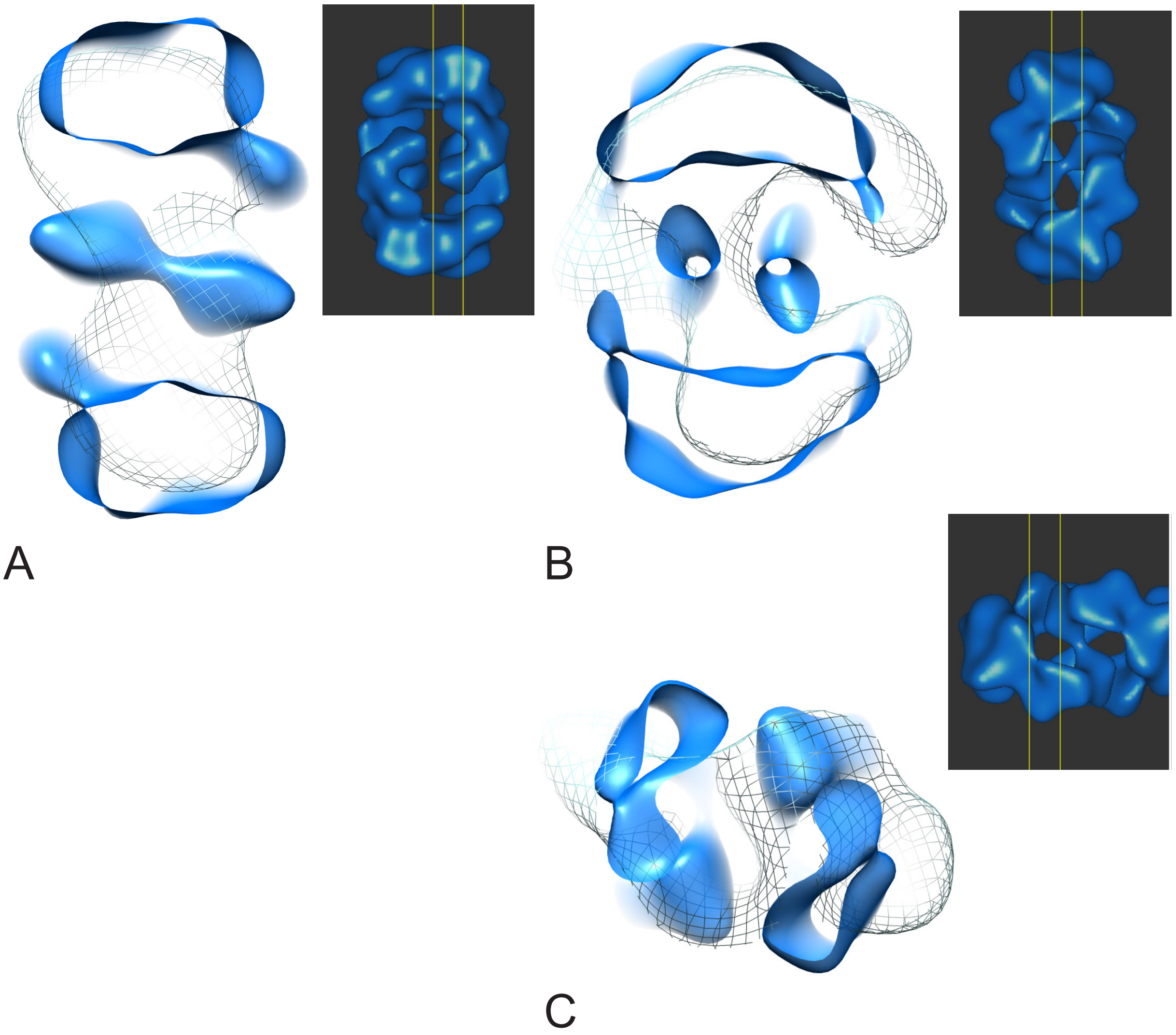

FIGURE S1. Comparison of the MUC5B D2 map with a reconstruction obtained without imposed symmetry (C1 map). Sections are shown with an approximative 20 Å thick-ness through the two maps following alignment with maximum overlap. The D2 map is shown surface rendered, while the low pass filtered C1 map is depicted with mesh representation. The views are along the two-fold axes from the side (a and b) and from the top (c) with slices at the positions indicated in the insets. Due to the lack of symmetry the C1 map was generated with wider angular steps than the corresponding map with imposed D2 symmetry. The first 3D-refinement was performed with an angular step of 13 degrees. The second refinement was performed with an angular step of 10 degrees, using the final map from the first refinement as input. The obtained resolution was 30 Å. The resolution of the map was estimated using the three-dimensional Fourier shell correlation (FSC) with a threshold of 0.143. The final map was low pass filtered to 50Å resolution.

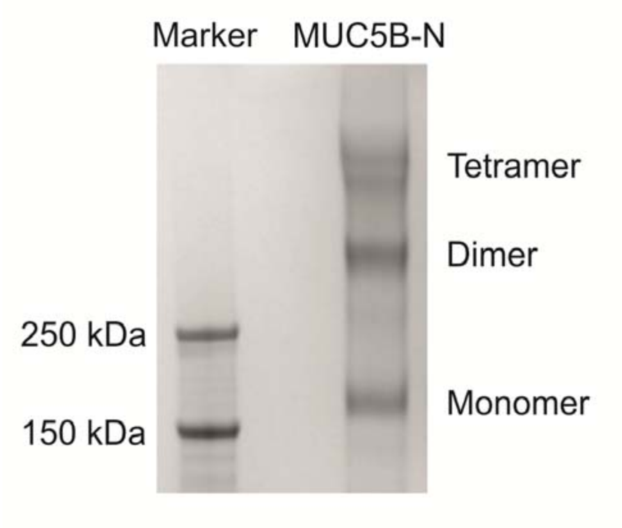

FIGURE S2. Glutaraldehyde-induced cross-link in the MUC5B-N tetramer. SDS-PAGE of cross-linked MUC5B-N stained by Commassie blue under reducing conditions.

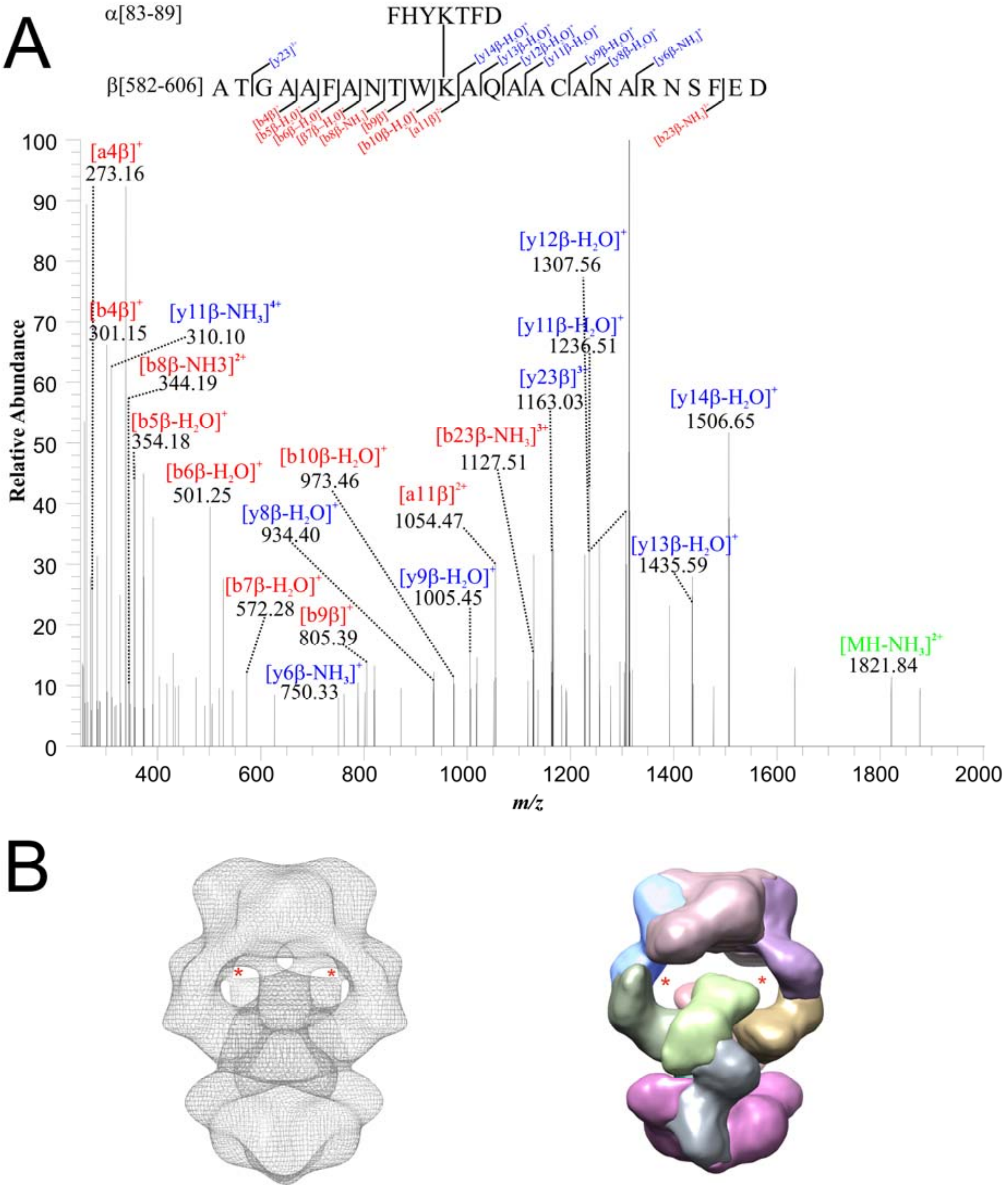

FIGURE S3. Glutaraldehyde-induced cross-link in the MUC5B-N tetramer. (A) Annotated MS2 fragment spectrum of the parent ion $[M+4H]^{4+}$ 915.66 for the glutaraldehyde cross-link between lysine 86 and lysine 592 of the MUC5B sequence. The mass deviation of the observed mass to the theoretical mass differed by -1.9 ppm. The α-peptide comprises amino acids 83 to 89 and the β-peptide residues 582 to 606 of the MUC5B N-terminus. Y ions are labelled in *blue*; b and a ions are labelled in red and the parent ion in green. (B) 3D density map of MUC5B-N oligomer. The mesh representation (left) at threshold value 0.9 illustrates the contacts between non-covalent dimers. The surface representation (right) shows in different colors the domains forming the MUC5B-N tetramer. Red stars mark the intermolecular D1-D2 interactions applicable to the glutaraldehyde cross-link that is described in Fig. S3A.
